# Gut microbiota as modulators of obesity and overweight: a registry-based systematic review of clinical trial evidence

**DOI:** 10.3389/fnut.2026.1865785

**Published:** 2026-07-02

**Authors:** Bolun Jiao, Sheng Jiang

**Affiliations:** Department of Endocrinology, The First Affiliated Hospital of Xinjiang Medical University, Urumqi, China

**Keywords:** analysis of clinical trial landscape, fecal microbiota transplantation, metabolic diseases, prebiotics, probiotics

## Abstract

**Background:**

Background: Obesity is a global epidemic that remains inadequately addressed by healthcare systems. The gut microbiota offers a promising metabolic target, yet systematic reviews of clinical trials on microbiome modulators for obesity are scarce.

**Methods:**

Using the Trialtrove database (September 16, 2025), we performed a registry-based systematic review with the strategy: “(Disease: Obesity) AND (Mechanism: Microbiome modulator).” We included interventional trials targeting overweight/obese populations with defined microbiome-modulating mechanisms; observational and withdrawn/suspended trials were excluded. Extracted data covered phase, status, intervention type, sponsor, location, and participant characteristics. Descriptive analyses used R software (v4.4.3).

**Results:**

Among 217 included trials, 131 (60%) were completed and 37 (17%) ongoing., Academic institutions led sponsorship (157 trials), followed by commercial (45) and government (14). Trials rose sharply after 2011, peaking at 34 in 2023 (over 80% of Phase IV trials that year). Probiotics dominated (141 trials), followed by synbiotics (21) and FMT (22). China (52) and the US (24) led research. Probiotics prevailed in Phases III/IV, whereas FMT concentrated in Phases II/IV with a higher termination rate.

**Conclusions:**

This study reveals a rapidly growing yet uneven landscape. Probiotics remain the primary focus, academic institutions the main sponsors, and China/US the core hubs. The field has entered a post-marketing evaluation phase dominated by Phase IV studies. Limitations include reliance on a single database and lack of efficacy data, but the study highlights rapid expansion and heterogeneity in this field. Future research should integrate multiple data sources and quality assessments for more comprehensive evidence.

## Introduction

Obesity has evolved from a simple metabolic disorder into a global epidemic and has become a major contributor to non-communicable diseases (NCDs) (1). As of 2022, approximately 2.518 billion adults worldwide were overweight, among whom 890 million were classified as obese. The prevalence of obesity has nearly doubled or tripled, and this trend continues to escalate in most regions (2). According to data from the World Obesity Federation, elevated body mass index (BMI ≥ 25 kg/m^2^) contributed to more than 3.6 million NCD-related deaths in 2023. Obesity constitutes a major risk factor for type 2 diabetes, metabolic dysfunction-associated steatotic liver disease, cardiovascular disease, hypertension, and at least 13 types of cancer. Meanwhile, the World Obesity Atlas projects that, without systematic intervention, the rising obesity rate will impose a heavier burden on cardiovascular diseases, diabetes, and musculoskeletal disorders; however, only 7% of countries currently possess well-prepared healthcare systems capable of addressing obesity (3). As the burden of obesity escalates while healthcare systems struggle to cope, the growing gap between these two realities underscores the urgent need for innovative, scalable, and mechanism-based therapeutic strategies. To bridge this gap, we conducted a comprehensive review of global clinical trials to map the research activity, geographical distribution, and temporal trends of microbiome-based interventions for obesity, rather than to quantitatively synthesize their efficacy.

The human gastrointestinal tract harbors a complex and dynamic microbial community—the gut microbiota, which forms a mutually beneficial symbiotic link with the host. The gut microbiota is increasingly regarded as a “metabolic organ” capable of regulating host energy homeostasis, systemic inflammation, and metabolic signaling (4–6). Germ-free mice are resistant to diet-induced obesity, and this phenotype can be reversed by fecal microbiota transplantation (FMT) from obese donors, indicating a causal role of gut microbiota in the pathogenesis of obesity (7, 8). Mechanistically, gut dysbiosis promotes obesity through multiple pathways, including reducing short-chain fatty acid (SCFA) production (compromising intestinal barrier integrity and GLP-1 secretion) (9, 10), disrupting bile acid metabolism (reducing brown adipose tissue thermogenesis) (11), and inducing chronic low-grade inflammation that exacerbates insulin resistance (12, 13). Together, these findings establish gut microbiota as both a pathogenic driver of obesity and a promising therapeutic target.

Microbiome-modulating therapies primarily involve a variety of strategies, each with its own unique mechanisms and clinical value. Probiotics are live microorganisms, such as *Lactobacillus* and *Bifidobacterium*, that confer health benefits to the host when administered in appropriate doses. They improve metabolism through various mechanisms, including competitive suppression of pathogenic bacteria, enhancement of intestinal barrier function, and production of SCFAs (13). A randomized double-blind controlled trial published in 2025 confirmed that *Bifidobacterium longum BL21* significantly reduced triglyceride levels in overweight and obese individuals and modulated the β-diversity of the gut microbiota (14). Prebiotics, such as fructooligosaccharides and inulin, selectively stimulate the growth of beneficial bacteria and the proliferation of beneficial microbes, thereby indirectly promoting health benefits. Synbiotics are combination formulations of probiotics and prebiotics designed to improve the survival and colonization efficiency of probiotics. Clinical studies have demonstrated their potential in reducing body fat and improving insulin sensitivity (15, 16). Postbiotics, a major focus of recent research, represent preparations of inactivated microorganisms or their components. Compared with live bacteria, postbiotics offer greater safety, stability, and storage convenience, and are less likely to contribute to antimicrobial resistance (17). FMT, by transferring the complete microbiome from healthy donors to recipients, can restore the intestinal microecology. Studies have shown that obese patients receiving FMT experience an average weight loss of 5%–10% and improvements in metabolic indicators. Live biotherapeutic products (LBPs) represent a new generation of more precise intervention strategies. For example, engineered bacteria targeting specific metabolic pathways or carefully selected next-generation probiotics (such as *Akkermansia muciniphila*) can function by regulating the microbiota–mitochondria axis and improving white adipose tissue function (18, 19). These microbiome-based therapies demonstrate significant potential at multiple levels for treating obesity by modulating the gut microbiota and provide new avenues for personalized, precision nutrition interventions.

Although extensive preclinical evidence has established gut microbiota as a potential therapeutic target for obesity, clinical translation of gut microbiota modulators has progressed unevenly. An increasing number of interventional trials have been initiated worldwide. Despite robust preclinical evidence, the major characteristics and patterns of existing global clinical evidence—regarding intervention types, geographic distribution, and temporal evolution—remain unclear. Notably, although previous narrative reviews have summarized the role of gut microbiota in obesity, there is still no systematic, quantitative, comprehensive review of global clinical trials on microbiome modulators targeting obesity across all intervention categories. The landscape analysis of clinical trials has gradually emerged as an important method for addressing such issues. Therefore, we conducted a landscape analysis of clinical trials by retrieving and organizing raw data from global clinical trials in the Trialtrove database.

## Methods

**FIGURE 1:**
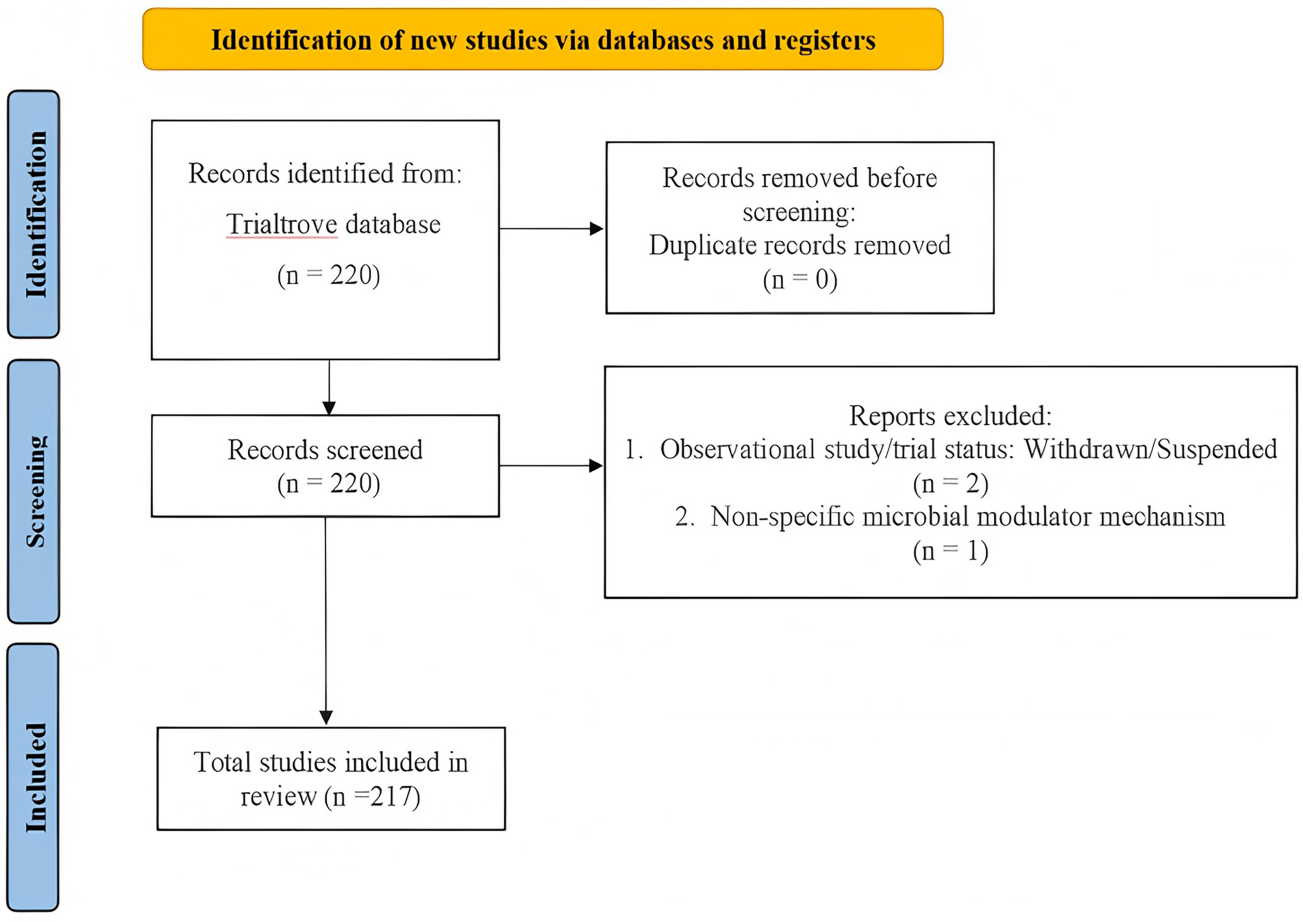
PRISMA flow diagram of trial selection.

We comprehensively searched the Trialtrove database^1^, an authoritative clinical trial database that integrates clinical trial data from diverse countries and regions worldwide, including ClinicalTrials.gov and WHO registries., As of September 16, 2025, the following search strategy was utilized: “(Disease is Metabolic/Endocrinology: Obesity) AND (Mechanism Of Action is Microbiome modulator).” Interventional clinical trials with explicit microbiome modulator mechanisms were included, with no restriction on study phase; observational studies, trials with a status of terminated or on hold, and trials not primarily targeting overweight/obese populations were excluded. Duplicate records were removed using the unique Trialtrove trial ID, with manual cross-checking of protocols/trial IDs and sponsor information when necessary. Interventions were primarily classified based on the Trialtrove database fields Primary Investigational Drug: Mechanism of Action and Primary Investigational Drug: Therapeutic Category. Probiotics were defined as live microorganisms (such as *Lactobacillus* and *Bifidobacterium*), and prebiotics were defined as substrates selectively utilized by host microorganisms (such as polysaccharides and oligosaccharides); when used together, they were considered synbiotics. FMT was identified through mechanisms such as “microbiome modulators, feces.” Cases with unclear classification were tackled through discussion between two researchers. Both researchers independently reviewed and verified the raw data, including extracting unstructured data, handling missing information, and addressing classification biases. Given the descriptive overview nature of this study, quality or risk-of-bias assessment was not conducted for each trial, in line with the standards for such studies. Data extraction and management were performed using Excel (version 16.0), and statistical analyses were made in R software (version 4.4.3). Since descriptive statistics (frequency, percentage, counts) were used, inferential statistical tests were not applied. Figures and tables were visualized using R 4.4.3 (with ggplot2, dplyr) and WebStorm 2025.3.3. Finally, we identified 217 clinical trials from the Trialtrove database (Figure 1). To ensure the transparency and methodological rigor of this analysis, we reported data sources, search strategies, inclusion criteria, data extraction, quality control, and descriptive analysis methods in accordance with the TITAN Guidelines 2025. These guidelines provide a standardized reporting framework for registry-based studies, effectively reducing methodological heterogeneity and quality gaps in the field (20). Detailed research content can be found in the Supplementary Appendix Table 1.

## Results

**FIGURE 2:**
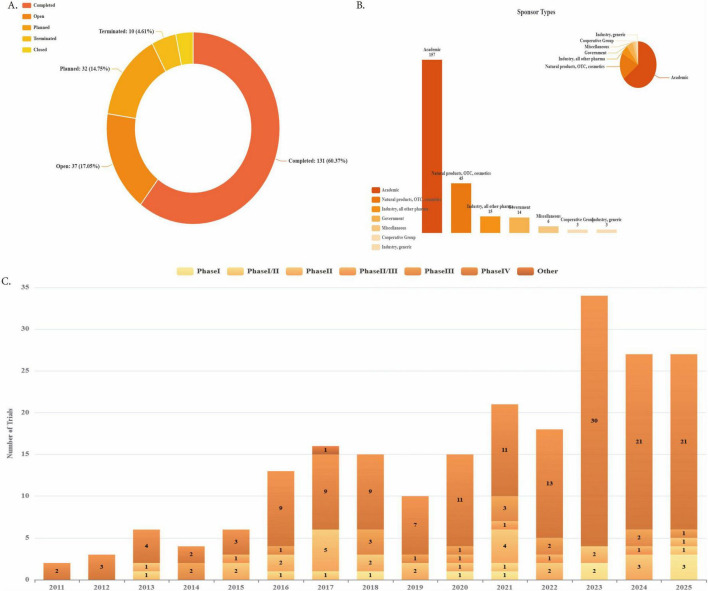
Multi-dimensional data analysis in clinical trials. **(A)** Distribution of clinical trial statuses. “Completed” = finished all procedures; “Ongoing” = recruiting/treating but not concluded; “Planned” = registered but not started; “Terminated” = stopped early without resumption; “Closed” = no new participants, possibly in follow-up/analysis. **(B)** Types of clinical trial sponsors. “Academic” = universities, hospitals, non-profits; “Commercial” = natural product, OTC, cosmetic, pharmaceutical companies; “Government” = national/regional agencies. **(C)** Distribution by year and trial phase. “Phase I” = small-cohort safety/tolerability/pharmacokinetics; “Phase II” = larger-group preliminary efficacy/safety; “Phase III” = large-scale confirmatory vs. standard/placebo; “Phase IV” = post-marketing long-term safety/effectiveness/optimal use.

This review included 217 clinical trials. Regarding trial status, 131 studies had been completed, accounting for 60% of the total, and 37 were ongoing open-label clinical trials, representing 17.05% (Figure 2A). Concerning funding sources, universities, hospitals, medical schools, and academic institutions contributed the largest share to the development of gut microbiome modulators (157 trials), followed by natural products, Over-The-Counter (OTC), cosmetics, pharmaceutical, and other commercial enterprises (45 trials); 14 trials received support from government agencies (Figure 2B). As for temporal distribution, only 2 trials were registered before 2011. The number was then increased significantly, reaching 34 trials in 2023, and maintained at a high level, with 27 trials in both 2024 and 2025. Notably, among Phase I to IV clinical trials, the proportion of Phase IV trials steadily increased over time, reaching 30 trials in 2023, exceeding 80% of the total trials for that year (Figure 2C).

**FIGURE 3:**
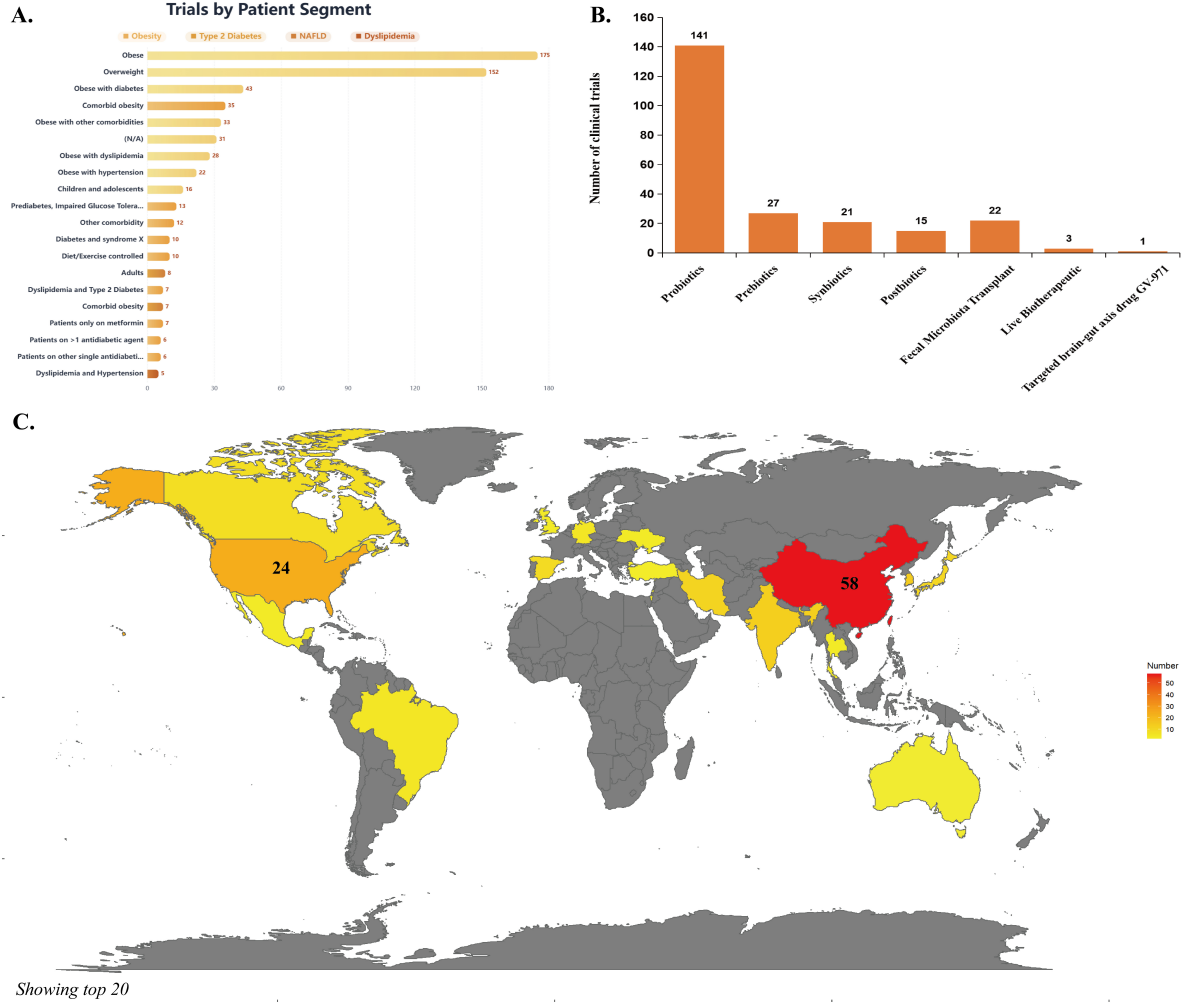
Multi-dimensional data analysis in clinical trials. **(A)** Patient disease type. “Obesity alone” = overweight/obese without other metabolic diseases; “Obesity + DM” = includes both obesity and diabetes; “Obesity + NAFLD” = includes both obesity and NAFLD; “Obesity + dyslipidemia” = includes both obesity and abnormal blood lipids; “Obesity + other comorbidities” = obesity with hypertension, CVD, or metabolic syndrome. **(B)** Microbiome modulator treatments. “Single-strain probiotics” = one live strain (e.g., *Lactobacillus*); “Multi-strain probiotics” = two or more live strains; “Prebiotics” = substrates stimulating beneficial bacteria (e.g., inulin, FOS); “Synbiotics” = probiotics + prebiotics; “FMT” = transfer of complete gut microbiota from healthy donor; “Postbiotics” = inactivated microorganisms or their metabolites; “LBPs” = engineered or selected bacteria for targeted therapy. **(C)** Geographic distribution. Shading intensity indicates the number of registered trials (darker = more trials).

Regarding participant characteristics, obesity-related studies encompassed multiple age groups, including children, adults, and older individuals. A large number of trials also included participants with comorbidities, such as diabetes (DM), non-alcoholic fatty liver disease (NAFLD), and dyslipidemia (Figure 3A). In terms of interventions, probiotics remained the primary focus of research (141 trials), whether as single strains or multi-strain formulations, applied alone or in combination. Trials involving prebiotic/probiotic combinations (synbiotics) (21 trials) and FMT (22 trials) also accounted for a considerable proportion (Figure 3B). As for geographical distribution, China (52 trials) and the United States (24 trials) led in the number of trials, followed by South Korea (14 trials), India (13 trials), Iran (11 trials), Japan (10 trials), Germany (5 trials), and the United Kingdom (4 trials) (Figure 3C). This distribution trend indicates that the regional development of microbiome-based therapies is closely linked to population density, national economic level, healthcare resource allocation, and research investment.

**FIGURE 4:**
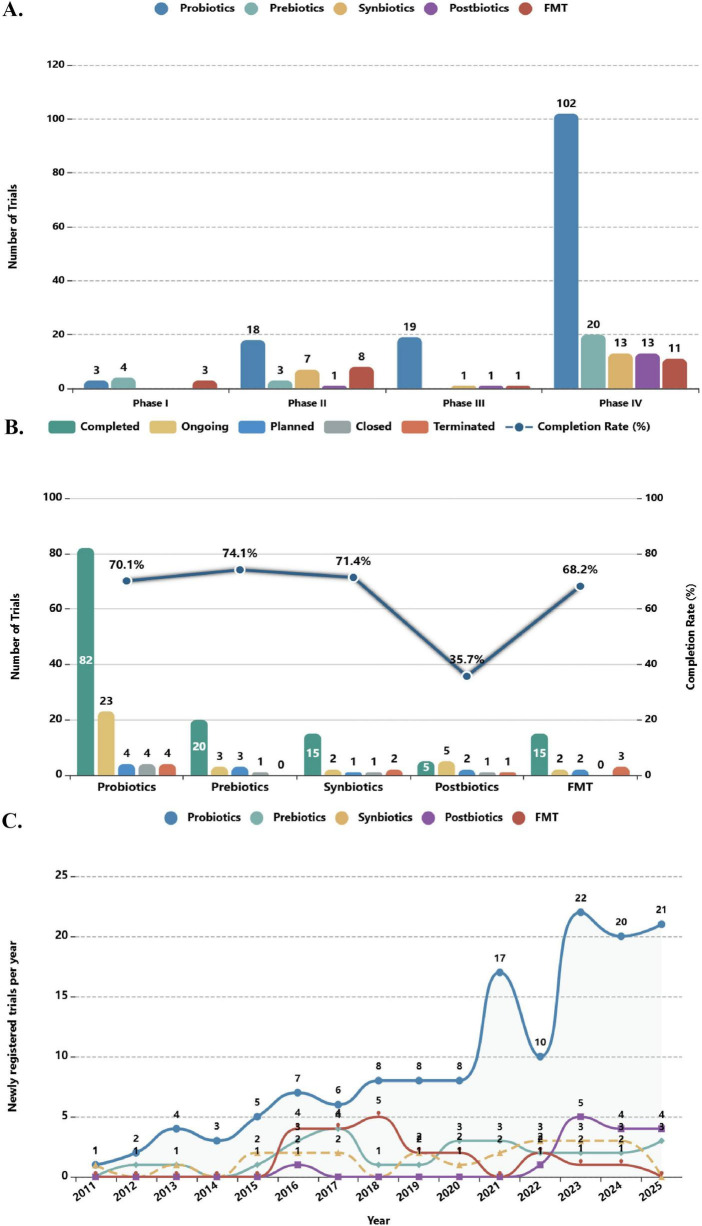
Subgroup analysis of clinical trials by intervention type. **(A)** Trial phase distribution: Probiotics dominate in Phase III (19 trials) and Phase IV (102 trials), while fecal microbiota transplantation (FMT) is concentrated in Phase II (8 trials) and Phase IV (11 trials). Synbiotics, postbiotics, and prebiotics remain in the exploratory stage. **(B)** Trial status and completion rates: Completion rate = completed / total registered; probiotics have the highest number of completions (82 trials), while FMT shows a relatively high termination rate. **(C)** Annual registration trends (2011–2025): Probiotics have steadily increased since 2011, peaking in 2023 (22 trials); FMT trials were more active during 2016–2019, reaching 5 trials in 2018; Postbiotics registrations have risen to 4–5 trials per year since 2022; Synbiotics and prebiotics maintain low but consistent annual registrations.

Finally, subgroup analysis by intervention type revealed differences in trial phase distribution, completion rates, and temporal trends (Figure 4). Probiotics dominated in Phase III (19 trials) and Phase IV (102 trials), while FMT was mainly concentrated in Phase II (8 trials) and Phase IV (11 trials) (Figure 4A). Trials about probiotics had the highest number of completed trials (82 trials), whereas FMT-related trials showed a higher termination rate (Figure 4B). Regarding temporal trends, the number of registered trials for probiotics steadily increased from 2011, peaking in 2023 (22 trials); the active period for FMT was concentrated between 2016 and 2019, peaking in 2018 (5 trials). Since 2022, the number of registered trials for postbiotics showed a moderate upward trend, reaching 4–5 per year; The annual number of registered trials for synbiotics and prebiotics remained relatively low but stable (Figure 4C).

## Discussion

A systematic analysis of 217 trials from the Trialtrove database provides the first structured and quantitative overview of gut microbiome modulators targeting obesity and overweight. The number of registered trials registered annually remained low until 2011, after which it increased sharply, peaking in 2023. This growth pattern aligns with the timeline of foundational discoveries that established the causal role of gut microbiota in obesity, including landmark germ-free mouse studies and the identification of specific microbial metabolites (21, 22). The accelerated development since 2011 indicates that there is a 12-year lag between the elucidation of mechanisms and clinical translation; this timeframe is comparable to that of other emerging therapeutic modalities (23). In addition, the majority of clinical trials were completed, and a considerable proportion were Phase IV trials, indicating that the field has moved from early exploration to post-marketing surveillance and real-world efficacy assessment. Academic institutions accounted for over 70% of the total research, suggesting that the field remains primarily driven by academia rather than dominated by major pharmaceutical companies. However, a recent systematic review and meta-analysis showed that microbiome interventions significantly improved waist circumference (MD: −1.62 cm) and body weight (MD: −1.50 kg) in patients with metabolic syndrome, but their effects on blood pressure and BMI did not reach statistical significance (24). Another meta-analysis on glucose and lipid metabolism reported similar findings: probiotic and synbiotic interventions consistently improved blood glucose and lipid profiles, whereas FMT showed heterogeneous results (25). These findings suggest that, despite the rapid increase in the number of trials, further research is needed to clarify the differences in efficacy among various intervention types.

The leading positions held by China and the United States reveal different research ecosystems. For China, this is closely linked to national strategic investments. The 14th 5-Year Plan highlights the bioeconomy as a key development area. The Expert Consensus on Gut Microecological Preparations and Fecal Microbiota Transplantation for Obesity, released in 2025, explicitly affirms the recommended role of probiotics and FMT (26). This consensus accelerates clinical adoption and fuels research through practice, thereby stimulating continuous evidence generation and creating a virtuous cycle of application and validation. In contrast, the United States is characterized by multi-level integration. As early as 2018, the National Institutes of Health (NIH) invested nearly $4 million in research on the link between gut microbial metabolites and health, laying the foundation for translational applications. In parallel, the FDA has progressively refined its regulatory framework for LBPs. This model ensures a clear translational pathway and a robust regulatory framework, striking a balance between safety and innovation.

Existing clinical studies provide supporting evidence for various microbiome interventions. In the field of probiotics, Wongsakorn et al. found that oral administration of probiotics (*Lactobacillus paracasei* MSMC39-1 and *Bifidobacterium animalis* TA-1) to patients with obesity and metabolic syndrome significantly reduced body weight, BMI, waist circumference, systolic blood pressure, and total cholesterol compared with the placebo group (27). Similarly, Guzailinuer et al. reported that supplementation with different doses of the novel probiotic strain *Lactobacillus paracasei* K56 effectively reduced body fat in adults with simple obesity (28). In the synbiotic field, Sanza et al. observed that after an 8-week intervention combining synbiotics with vitamin D, overweight/obese women experienced significant reductions in waist circumference, fat mass, body fat percentage, and visceral fat area compared with the placebo group (29). In the field of prebiotics, Rahele and colleagues found that, compared with a placebo group, administration of prebiotics—mainly inulin-type fructans—to overweight or obese women with polycystic ovary syndrome significantly improved their body weight, fasting insulin, HOMA-IR, and insulin sensitivity (30). In addition, recent studies on the novel Alzheimer’s drug GV-971 reported that it improved cognitive function and alleviated neuropsychiatric symptoms by modulating the gut microbiota (31, 32). Although this finding does not directly address obesity, it provides new insights into the potential application of gut microbiota in central metabolic regulation.

Fateme et al. found that for patients undergoing weight-loss surgery, incorporating probiotic supplementation into a weight-loss program combined with cognitive behavioral therapy was more effective than the weight-loss program and cognitive behavioral therapy alone in reducing food addiction, preventing weight regain, and improving eating behaviors (33). However, a recent Cochrane systematic review of 17 studies (838 participants) evaluated the effects of probiotics, prebiotics, synbiotics, SCFAs, and FMT on obesity management in children and adolescents. The review concluded that the quality of evidence was very low, and in adolescents (ages 10–19), probiotics may have little or no effect on BMI, weight, waist circumference, and other outcomes (34). Collectively, although several single-center studies report positive results, overall clinical evidence remains limited, especially in the pediatric population.

Our subgroup analysis revealed distinct translational patterns among various intervention types. Trials involving probiotics have transitioned from early exploratory phases to post-marketing surveillance phases, with Phase IV trials far outnumbering those in earlier phases; however, recent studies indicated that the metabolic benefits of *Akkermansia muciniphila* supplementation depended largely on baseline gut levels, with significant reductions in body weight, fat mass, and HbA1c observed only in individuals with lower baseline levels (35). This finding suggests that even in the relatively established field of probiotics, future approaches should shift from a “one-size-fits-all” approach to precision interventions based on individual gut microbiome profiles. In contrast, most FMT trials were concentrated in Phases II and IV, with relatively high termination rates. Global surveys showed significant disparities in FMT accessibility across countries, primarily due to inadequate infrastructure, funding limitations, and regulatory uncertainties (36). There are considerable differences in FMT classifications across countries. In the United States, the FDA classifies it as a drug and biological product; the UK considers it a medicine, the European Union categorizes it as a human-derived material under the Human Tissue Regulation, and Australia lists it as a biological product. This inconsistency in regulatory standards directly hinders the standardization and clinical adoption of FMT (37–39). Furthermore, although the FDA has approved two FMT products (Rebyota and Vowst) for the treatment of recurrent *Clostridioides difficile* infection, their high costs and limited insurance coverage have hindered investment (16). Notably, since 2022, the increasing number of postbiotic trials indicates that research is shifting toward new strategies. As inactivated microbial preparations, postbiotics offer greater safety (no colonization risk) and stability than live probiotics (40). The European Union has granted a novel food authorization for pasteurized *Akkermansia muciniphila*, representing a significant regulatory landmark (41). The global market for postbiotic supplements is estimated to reach $12.9 billion by 2034 (42). However, the field of postbiotics still faces challenges, such as a lack of standardization, limited strain-specific efficacy, poor clinical reproducibility, and the need for greater coordination of global regulatory frameworks. Inconsistent results often arise when translating results from animal models to clinical applications, highlighting the necessity of precise functional analysis and multi-omics integration (16). In summary, the gap in translational research is not merely a matter of time but is fundamentally influenced by the specific characteristics of the interventions. Bridging this gap requires tailored strategies based on the features of different intervention types.

### Research limitations

Several limitations should be acknowledged. First, our analysis is based on a single database (Trialtrove), which may not comprehensively capture regional trials from all regions; even among registered trials, key variables, such as primary outcomes and intervention details, are often not fully reported. Second, interventions are classified based on information provided by study sponsors, which may result in heterogeneity across trials. Third, our analysis does not include trial outcomes or efficacy data; therefore, the patterns we describe reflect trends in research activity and investment rather than therapeutic effectiveness. Fourth, unstructured data remain susceptible to interpretive bias despite double-checking. Fifth, as a landscape analysis, we do not assess quality or risk-of-bias for individual trials. Sixth, publication bias may affect representativeness, particularly for negative or null results. Seventh, discrepancies between registered and completed outcomes (outcome reporting bias) may distort the interpretation of trial activity and research priorities. Our analysis does not systematically compare registered protocols with published results, and this should be considered.

### Future direction

Based on the patterns observed in these 217 trials, future research can focus on three directions. First, future research can improve the evidence system and evaluation standards. Currently, trials on probiotics dominate, but existing studies show high heterogeneity in outcome measures, and the quality of existing evidence is low. Although there are numerous Phase IV trials, long-term data remain insufficient. It is recommended to establish a set of standardized core outcome measures (covering body weight, waist circumference, blood glucose, microbiota composition, and SCFAs), prioritize funding for randomized controlled trials with sufficient sample sizes and long durations, and leverage existing Phase IV trial platforms to systematically collect long-term safety and efficacy data. Second, efforts should be made to promote global multicenter collaborative validation. Studies in China and the United States account for a high proportion, indicating significant regional concentration. It is necessary to establish international collaborative networks to validate interventions across populations with different genetic backgrounds, dietary habits, and baseline gut microbiota compositions. Third, future research should facilitate the clinical translation of academic findings. Funding from academic institutions remains relatively high, while investment from major pharmaceutical companies is limited. It is recommended that the academic community collaborate with regulatory agencies such as the FDA to clarify the registration pathway for microbiome-based therapeutics targeting metabolic diseases, including the specific requirements and approval processes for transitioning from dietary supplements to LBPs.

## Conclusion

This study demonstrates that the number of clinical trials on gut microbiota modulators for obesity and overweight is growing rapidly, but there remains an uneven distribution in intervention types, sponsors, and geographic regions. Over 60% of the trials have been completed, with Phase IV studies dominating, particularly in the probiotic field, where most trials have advanced to the post-marketing evaluation phase. Academic institutions are the primary sponsors, while China and the United States are the main research hubs. Probiotics continue to dominate the intervention type; although FMT experienced a period of active research between 2016 and 2019, it shows a relatively high termination rate. Postbiotic trials have shown moderate growth since 2022. Although this review is limited by its reliance on a single database and the absence of efficacy or quality assessments, it provides the first systematic, quantitative overview of the global clinical trials in this field. Future analyses should integrate registry data from multiple sources and include formal quality and risk-of-bias assessments to better inform research prioritization and clinical translation.

## Data Availability

The original contributions presented in this study are included in this article/supplementary material, further inquiries can be directed to the corresponding author.

## Footnotes

1
https://clinicalintelligence.citeline.com

## References

[1] Hildebrand S Pfeifer A. The obesity pandemic and its impact on non-communicable disease burden. *Pflugers Archiv* (2025) 477:657–68. 10.1007/s00424-025-03066-8 39924587 PMC12003543

[2] NCD Risk Factor Collaboration (NCD-RisC). Worldwide trends in underweight and obesity from 1990 to 2022: a pooled analysis of 3663 population-representative studies with 222 million children, adolescents, and adults. *Lancet.* (2024) 403:1027–50. 10.1016/S0140-6736(23)02750-2 38432237 PMC7615769

[3] World Obesity Federation,. *World Obesity Atlas 2025.* (2025). Available online at: https://www.worldobesity.org/resources/resource-library/world-obesity-atlas-2025 (accessed April 2, 2026).

[4] Van Hul M Neyrinck AM Everard A Abot A Bindels LB Delzenne NM et al. Role of the intestinal microbiota in contributing to weight disorders and associated comorbidities. *Clin Microbiol Rev* (2024) 37:e4523. 10.1128/cmr.00045-23 38940505 PMC11391702

[5] Dimeji IY Ayodeji AS. Pharmacological modulation of the gut microbiota and endotoxemia: a next-generation approach to treating metabolic syndrome. *Aspet Discov.* (2025) 1:100010. 10.1016/j.aspetd.2025.100010

[6] Lin X Yu Z Liu Y Li C Hu H Hu J et al. Gut-x axis. *Imeta* (2025) 4:e270. 10.1002/imt2.270 40027477 PMC11865426

[7] Bäckhed F Manchester JK Semenkovich CF Gordon JI. Mechanisms underlying the resistance to diet-induced obesity in germ-free mice. *Proc Natl Acad Sci U S A* (2007) 104:979–84. 10.1073/pnas.0605374104 17210919 PMC1764762

[8] Ridaura VK Faith JJ Rey FE Cheng J Duncan AE Kau AL et al. Gut microbiota from twins discordant for obesity modulate metabolism in mice. *Science* (2013) 341:1241214. 10.1126/science.1241214 24009397 PMC3829625

[9] Yang-Jensen SK Nägele NS Jensen BAH. From gut to blood: barrier dysfunction as a driver of systemic low-grade inflammation in cardiometabolic disease. *Am J Physiol Cell Physiol.* (2025) 329:C1723–41. 10.1152/ajpcell.00704.2025 41138203

[10] Toft PB Yashiro H Erion DM Gillum MP Bäckhed F Arora T. Microbial dietary protein metabolism regulates glp-1 mediated intestinal transit. *FASEB J* (2023) 37:e23201. 10.1096/fj.202300982R 37732618

[11] Ma H Wu Y Li D Sun H Xie Y Zhao S et al. Gut microbiota drives the metabolic dysregulation in obesity-prone individuals by impairing GDCA-mediated activation of brown adipose thermogenesis and ileal GLP-1 secretion. *Acta Pharm Sin B.* (2026) 16:836–53. 10.1016/j.apsb.2025.12.006 41685140 PMC12891870

[12] Patterson E Ryan PM Cryan JF Dinan TG Ross RP Fitzgerald GF et al. Gut microbiota, obesity and diabetes. *Postgrad Med J* (2016) 92:286–300. 10.1136/postgradmedj-2015-133285 26912499

[13] Ngabea MA Dimeji IY. GLP-1 receptor agonists and inflammatory pathway modulation: dual targeting of metabolic and immune dysfunction in insulin resistance. *Biochem Biophys Res Commun* (2025) 789:152822. 10.1016/j.bbrc.2025.152822 41161090

[14] Pires L González-Paramás AM Heleno SA Calhelha RC. Exploring therapeutic advances: a comprehensive review of intestinal microbiota modulators. *Antibiotics* (2024) 13:720. 10.3390/antibiotics13080720 39200020 PMC11350912

[15] Wang X Xing Z Wang R Zhang G Liu G Li Z et al. Multi-omics analyses the effect of *Bifidobacterium longum* subsp. Longum BL21 supplementation on overweight and obese subjects: a randomized, double-blind, placebo-controlled study. *Nutr Metab* (2025) 22:79. 10.1186/s12986-025-00969-2 40676704 PMC12273011

[16] Zhang C Zhang Q Zhang X Du S Zhang Y Wang X et al. Effects of synbiotics surpass probiotics alone in improving type 2 diabetes mellitus: a randomized, double-blind, placebo-controlled trial. *Clinical Nutr* (2025) 44:248–58. 10.1016/j.clnu.2024.11.042 39719724

[17] Safavi M Farajian S Kelishadi R Mirlohi M Hashemipour M. The effects of synbiotic supplementation on some cardio-metabolic risk factors in overweight and obese children: a randomized triple-masked controlled trial. *Int J Food Sci Nutr* (2013) 64:687–93. 10.3109/09637486.2013.775224 23477506

[18] Ahmed N Gaur V Kamle M Chauhan A Chauhan R Kumar P et al. Microbiome-based therapeutics for metabolic disorders: harnessing microbial intrusions for treatment. *Front Med Technol.* (2025) 7:1695329. 10.3389/fmedt.2025.1695329 41246285 PMC12613280

[19] Zheng X Huang W Li Q Chen Y Wu L Dong Y et al. Membrane protein amuc_1100 derived from *Akkermansia muciniphila* facilitates lipolysis and browning *via* activating the AC3/PKA/HSL pathway. *Microbiol Spectr* (2023) 11:e432322. 10.1128/spectrum.04323-22 36847500 PMC10100790

[20] Agha R Mathew G Rashid R Kerwan A Al-Jabir A Sohrabi C et al. Transparency in the reporting of artificial intelligence – the titan guideline. *Prem J Sci.* (2025) 10:100082. 10.70389/PJS.100082

[21] Bäckhed F Ding H Wang T Hooper LV Koh GY Nagy A et al. The gut microbiota as an environmental factor that regulates fat storage. *Proc Natl Acad Sci U S A* (2004) 101:15718–23. 10.1073/pnas.0407076101 15505215 PMC524219

[22] Zhong W Wang H Yang Y Zhang Y Lai H Cheng Y et al. High-protein diet prevents fat mass increase after dieting by counteracting lactobacillus-enhanced lipid absorption. *Nat Metab* (2022) 4:1713–31. 10.1038/s42255-022-00687-6 36456724

[23] DeMaria A. Implementation: the final step in translating innovation. *Structural Heart* (2024) 8:100359. 10.1016/j.shj.2024.100359 39290677 PMC11403083

[24] Kaul R Paul P Ayyan M Harfouche M Laws S Chaari A. S774 the effect of gut microbiome-modulating therapies on anthropometric parameters and blood pressure in metabolic syndrome: a systematic review, meta-analysis and meta-regression. *Am J Gastroenterol.* (2025) 120:S166. 10.14309/01.ajg.0001130556.74421.96

[25] Mederle AL Dima M Stoicescu ER Cãpãstraru BF Levai CM Haţegan OA et al. Impact of gut microbiome interventions on glucose and lipid metabolism in metabolic diseases: a systematic review and meta-analysis. *Life* (2024) 14:1485. 10.3390/life14111485 39598283 PMC11595434

[26] Yuan HJ Yang JP Deng XR Heng H Liu Y Yang X. Consensus on intestinal microecological preparations and fecal microbiota transplantation in the treatment of obesity. *Chin J Pract Diag Ther.* (2025) 39:193–202.

[27] Luangphiphat W Prombutara P Jamjuree P Chantarangkul C Vitheejongjaroen P Muennarong C et al. The efficacy of *Lacticaseibacillus paracasei* MSMC39-1 and *Bifidobacterium animalis* TA-1 probiotics in modulating gut microbiota and reducing the risk of the characteristics of metabolic syndrome: a randomized, double-blinded, placebo-controlled study. *Plos One* (2025) 20:e317202. 10.1371/journal.pone.0317202 39792908 PMC11723615

[28] Kadeer G Fu W He Y Feng Y Liu W Hung W et al. Effect of different doses of *Lacticaseibacillus paracasei* K56 on body fat and metabolic parameters in adult individuals with obesity: a pilot study. *Nutr Metab* (2023) 20:16. 10.1186/s12986-023-00739-y 36944956 PMC10031870

[29] Jamshidi S Masoumi SJ Abiri B Sarbakhsh P Sarrafzadeh J Nasimi N et al. The effect of synbiotic and vitamin d co-supplementation on body composition and quality of life in middle-aged overweight and obese women: a randomized controlled trial. *Clin Nutr Espen* (2022) 52:270–6. 10.1016/j.clnesp.2022.09.005 36513465

[30] Ziaei R Shahshahan Z Ghasemi-Tehrani H Heidari Z Nehls MS Ghiasvand R. Inulin-type fructans with different degrees of polymerization improve insulin resistance, metabolic parameters, and hormonal status in overweight and obese women with polycystic ovary syndrome: a randomized double-blind, placebo-controlled clinical trial. *Food Sci Nutr* (2023) 12:2016–28. 10.1002/fsn3.3899 38455215 PMC10916604

[31] Wang X Xie Z Yuan J Jin E Lian W Chang S et al. Sodium oligomannate disrupts the adherence of rib(high) bacteria to gut epithelia to block SAA-triggered TH1 inflammation in 5XFAD transgenic mice. *Cell Discov* (2024) 10:115. 10.1038/s41421-024-00725-5 39557828 PMC11573985

[32] Wang X Sun G Feng T Zhang J Huang X Wang T et al. Sodium oligomannate therapeutically remodels gut microbiota and suppresses gut bacterial amino acids-shaped neuroinflammation to inhibit Alzheimer’s disease progression. *Cell Res* (2019) 29:787–803. 10.1038/s41422-019-0216-x 31488882 PMC6796854

[33] Ghafouri-Taleghani F Tafreshi AS Doost AH Tabesh M Abolhasani M Amini A et al. Effects of probiotic supplementation added to a weight loss program on anthropometric measures, body composition, eating behavior, and related hormone levels in patients with food addiction and weight regain after bariatric surgery: a randomized clinical trial. *Obes Surg* (2024) 34:3181–94. 10.1007/s11695-024-07437-5 39117856

[34] Fahim SM Huey SL Palma Molina XE Agarwal N Ridwan P Ji N et al. Gut microbiome-based interventions for the management of obesity in children and adolescents aged up to 19 years. *Cochrane Database Syst Rev.* (2025) 7:CD15875. 10.1002/14651858.CD015875 40637175 PMC12243456

[35] Zhang Y Liu R Chen Y Cao Z Liu C Bao R et al. *Akkermansia muciniphila* supplementation in patients with overweight/obese type 2 diabetes: efficacy depends on its baseline levels in the gut. *Cell Metab* (2025) 37:592–605. 10.1016/j.cmet.2024.12.010 39879980

[36] Khoruts A. The challenges and opportunities in the expanding horizons of microbiota transplant therapies. *Gut Microbes* (2025) 17:2559032. 10.1080/19490976.2025.2559032 41181931 PMC12584655

[37] Deepti I Chettri B Mehra A Pinheiro AM Ravi R. Faecal microbiota transplantation for recurrent *Clostridiodes difficile* infection & its global regulatory landscape. *Indian J Med Res* (2025) 161:113–9. 10.25259/IJMR_818_2024 40257135 PMC12010788

[38] Mikkelsen TA McIlroy JR Mimiague M Rouanet A Sterkman L. Towards an EU-wide suitable regulatory framework for faecally derived, industrially manufactured medicinal products. *Unit Eur Gastroenterol J* (2020) 8:351–2. 10.1177/2050640620910313 32213033 PMC7184666

[39] Ossorio PN Zhou Y. Regulating stool for microbiota transplantation. *Gut Microbes* (2019) 10:105–8. 10.1080/19490976.2018.1502537 30212271 PMC6546323

[40] Mokashe NU Talkal R Tokdar P. Postbiotics: definitions, manufacturing, analytical characterization, and clinical evidence for human and animal health. *Curr Microbiol* (2026) 83:278. 10.1007/s00284-026-04865-7 41931125

[41] European Commission. *Amends EU Novel Food Rules to Extend Use of Pasteurised Akkermansia muciniphila in Food Supplements and Foods for Special Medical Purposes for Adolescents. Regulation (EU) 2026/391.* (2026). Available online at: https://eur-lex.europa.eu/ (accessed June 5, 2026).

[42] Global Market Insights Inc. *Postbiotic Supplements Market - Size, Share & Growth Analysis 2025-2034. Report No. GMI7076.* (2025). Available online at: https://www.gminsights.com/industry-analysis/postbiotic-supplements-market (accessed June 5, 2026).

